# G against Glioma: G protein inhibitory α subunit 2 (Gαi2) as a novel glioma target

**DOI:** 10.7150/ijbs.82530

**Published:** 2023-01-22

**Authors:** Georgios S. Markopoulos

**Affiliations:** 1 Haematology Laboratory-Unit of Molecular Biology, University Hospital of Ioannina, Ioannina, Greece.; 2 Neurosurgical Institute, Faculty of Medicine, University of Ioannina, 45110 Ioannina, Greece.

## Abstract

Glioma is among the cancers with the highest mortality and morbidity. The complex biology and acquired chemoresistance create a continuous need for novel, effective therapies. In a recent report by Wang et al, an elegant bioinformatic analysis revealed potential functions and underlying chemopreventive mechanisms involving G protein inhibitory α subunit 2 (Gαi2), which activates NF-κB, a master transcription factor in the regulation of glioma. Given that NF-κB takes part in positive regulatory feedback loops during glioma development, Gαi2 is an attractive candidate for targeted therapies. This discovery unlocks new avenues towards understanding the biology of gliomagenesis as well as the discovery of novel targeted antiglioma agents.

## Introduction

High grade gliomas are among the most aggressive brain malignancies with a significantly low median survival of less than 15 months. With >300.000 new cases and >250.000 deaths, gliomas are considered as one of the deadliest types of cancer per case. The standard therapeutic protocols include surgical resection with concurrent radiotherapy and/or adjuvant chemotherapy. The latest WHO classification includes several molecular markers that reveal a complex biology which explain the acquired chemoresistance. Indeed, there is a continuous need for novel, effective therapies against this devastating disease 1.

Heterotrimeric G-proteins (with subunits α, β and γ) have been established as essential component of intracellular signal transduction with various roles in physiology and pathology. The different roles of each subunit as well as the discrete combination of trimers can lead to distinct physiological outcomes. G-protein coupled signaling is a universal cellular mechanism that mediates responses to a wide array of signaling molecules (such as photons, hormones and neurotrasmiters). G-proteins are primarily diversed based on the Gα subunit. Inhibitory α subunits, such as Gαi2 lead to cAMP depletion, through the inhibition of adenylic cyclase enzyme 2. Previous reports have implicated G protein coupled signalling in gliomagenesis 3. Gαi3 has been previously verified as an important factor for activation and proliferation of glioma cells, through induction of Akt/mTOR signalling pathway 4.

In this issue, the role of Gαi2 subunit in glioma has been report by Wang et al., published in International Journal of Biological Sciences 5. Utilizing a sophisticated bioinformatic analysis the authors have found that Gαi2 is overexpressed in glioma and is associated with a significant lower overall survival. Gαi2 expression has been also corelated with a higher tumor grade and a wild-type isocitrate dehydrogenase (IDH) status. Importantly, knockdown or knockout of Gαi2 in glioma cell lines resulted in a significant antiglioma effect, leading to reduced viability, proliferation and mobility, and an induction of apoptotic cell death. The authors confirmed their results with a series of *in vitro* experiments and in ex vivo xenografts.

The underlying mechanism proposed by the authors involved the activity of NF-κB, a master transcription factor in the regulation of glioma 1. Gαi2 downregulation led to NF-κB inhibition through p65 subunit dephosphorylation and reduced NF-κB target-genes expression. On the contrary, Sp1 transcription factor was found to be among those that mediate Gαi2 overexpression, since Sp1 has been verified as bound to Gαi2 promoter, following chromatin immunoprecipitation analysis. Sp1 may act synergistically with NF-κB to promote proliferation and migration of glioma cells 5.

**Figure 1:**
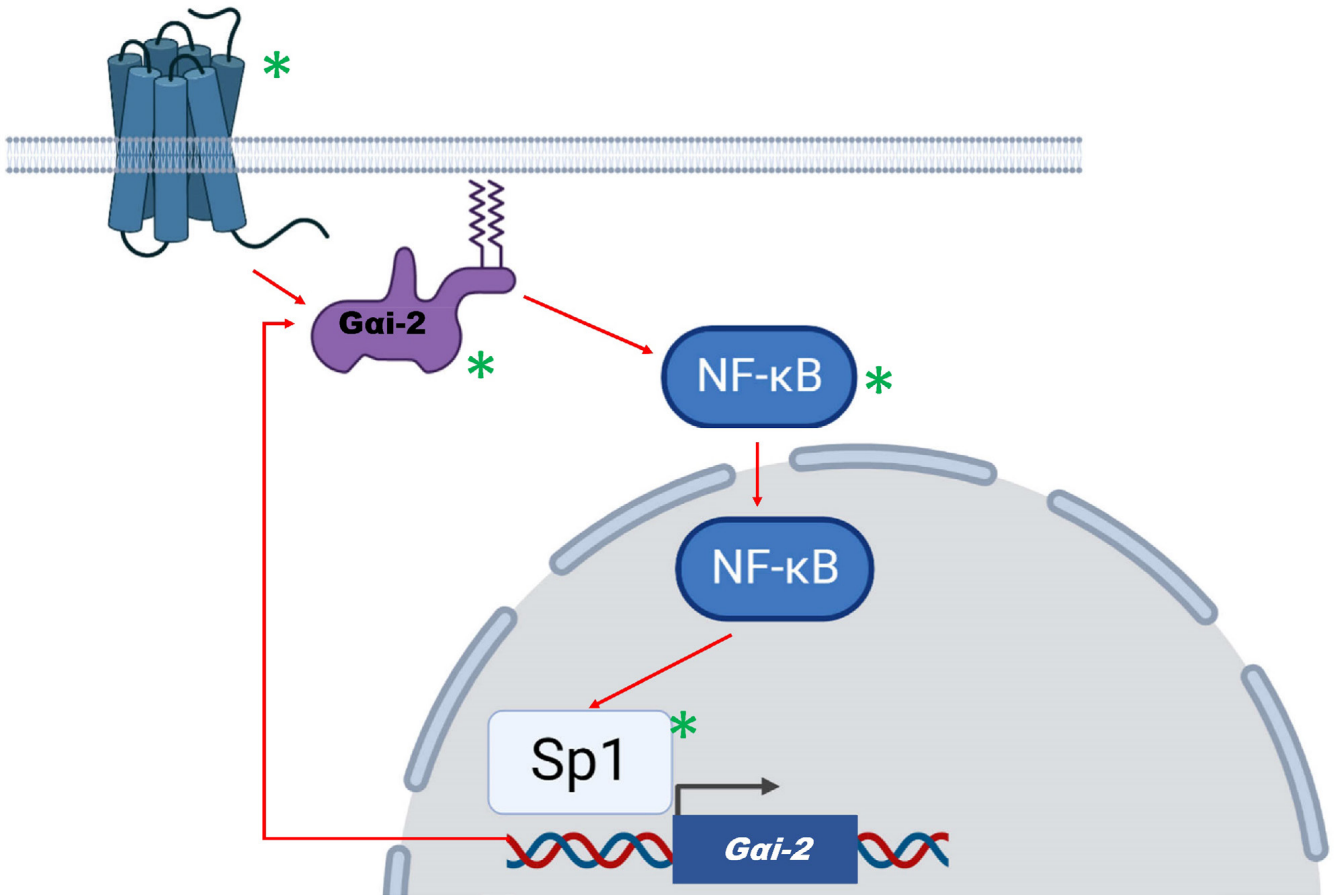
** A proposed model of action for Gαi2 in human glioma.** Gαi2 can be activated by extrinsic (G protein coupled receptors activation) or intrinsic (oncogenic signals) factors. Based on Wang et al, Gαi2 activates NF-κB which in turn may assist in Sp1-mediated Gαi2 upregulation. Thus, a positive feedback loop is formed that maintains gliomagenesis. Possible molecular targets that are involved in this mechanism are denoted by a green asterisk.

Taken together, available data support that the activation/expression of Gαi2 may contribute towards gliomagenesis through a possible feedback mechanism that involves the activation of NF-κB and Sp1 (Figure 1). In the proposed mechanism, Gαi2 is activated either by GPCR receptor mediated extrinsic signallling, or through oncogene-induced intrinsic mechanisms. The resulting NF-κB activation promotes gliomagenesis and further enhances Gαi2 expression and activation by acting co-operatively with Sp1 factor. GPCRs, Gαi2, NF-κB and Sp1 are potential molecular targets that would disrupt this feedback-loop mechanism. A similar mechanism has been proposed by our team, involving the activation of NF-κB by several cluster of differentiation (CD) molecules that act as glioma markers 1, 6. Targeting Gαi2 along with agents with a potent action against NF-κB, such as the natural product deglucohellebrin 7 would be a promising therapeutic strategy.

## Conclusions and future perspectives

Given the nature of central nervous system malignancies and the low median survival of glioma, there is a continuous need for new therapeutic approaches. The influential study by Wang et al. puts Gαi2 in the map of available targets for glioma and suggest its involvement in feedback loops that drive gliomagenesis (Figure 1) 5. This novel conceptual framework unlocks new avenues towards understanding the molecular mechanisms that drive gliomagenesis. Thus, it may assist in the discovery of novel targeted therapies against one of the most aggressive types of malignancy.
